# Prognostic Factor Utility of BAP1 Immunohistochemistry in Uveal Melanoma: A Single Center Study in Spain

**DOI:** 10.3390/cancers13215347

**Published:** 2021-10-25

**Authors:** Laura Tabuenca Del Barrio, Luiz Miguel Nova-Camacho, Alicia Zubicoa Enériz, Iñigo Martínez de Espronceda Ezquerro, Alicia Córdoba Iturriagagoitia, Enrique Borque Rodríguez-Maimón, Alfredo García-Layana

**Affiliations:** 1 Complejo Hospitalario de Navarra, Department of Ophthalmology, Calle Irunlarrea s/n, 31008 Pam-plona, Spain; alicia.zubicoa.eneriz@navarra.es (A.Z.E.); enrique.borque.rodriguez@navarra.es (E.B.R.-M.); 2 IdiSNA, Navarra Institute for Health Research, Calle Irunlarrea s/n, 31008 Pamplona, Spain; imezquerro@riojasalud.es (I.M.d.E.E.); aglayana@unav.es (A.G.-L.); 3 Complejo Hospitalario de Navarra, Department of Pathology, Calle Irunlarrea s/n, 31008 Pamplo-na, Spain; luizmiguel.novacamacho@osakidetza.eus (L.M.N.-C.); alicia.cordoba.iturriagagoitia@navarra.es (A.C.I.); 4 Complejo Hospitalario de Navarra, Department of Dermatology, Calle Irunlarrea s/n, 31008 Pamplo-na, Spain; 5 Department of Ophthalmology, Clínica Universidad de Navarra, Avenida de Pio XII 36, 31008 Pam-plona, Spain; 6 Instituto de Salud Carlos III, 28029 Madrid, Spain

**Keywords:** uveal melanoma, metastasis, survival, BAP1 immunohistochemical stain, prognostic factors

## Abstract

**Simple Summary:**

As uveal melanoma metastasis rates are still very high, the mechanisms by which it spreads need to be evaluated. Our research sought to determine which pathological and clinical features were correlated with the prognosis of uveal melanoma in a Spanish community. BAP1 (BRCA1-Associated Protein 1) gene mutation is one of the strongest predictors for metastasis in uveal melanoma. The BAP1 protein has a tumor suppressor function and the presence of the BAP1 protein can be shown using immunohistochemical staining. Our study showed that nuclear BAP1 immunostaining had a significant correlation with survival rate in our sample, and patients with a lack of nuclear BAP1 immunostaining should be considered high-risk and receive a close follow-up. This stain can be used as routine technique in the pathological examination of uveal melanoma.

**Abstract:**

Even today, the mortality rate for uveal melanoma (UM) remains very high. In our research, we sought to determine which pathological and clinical features were correlated with the prognosis of UM. BAP1 (BRCA1-Associated Protein 1) gene mutation has been analyzed as one of the strongest predictors for metastasis in UM. The BAP1 gene codifies the BAP1 protein which has a tumor suppressor function. The presence of this protein can be determined by BAP1 immunohistochemical staining. Eighty-four uveal melanoma patients and forty enucleated eyeballs were examined. Metastasis was present in 24 patients. Nuclear BAP1 staining was low in 23 patients. The presence of a higher large basal diameter tumor (*p* < 0.001), tumor infiltrating lymphocytes (*p* = 0.020), and a lack of nuclear BAP1 immunostaining (*p* = 0.001) ocurred significantly more often in the metastatic group. Metastasis-free survival was lower in patients with low nuclear BAP1 staining (*p* = 0.003). In conclusion, to the best of our knowledge, this is the first time that BAP1 staining has been studied in uveal melanoma in a Spanish community. We believe that this technique should become routine in the pathological examination of uveal melanoma in order to allow adequate classification of patients and to establish an individual follow-up plan.

## 1. Introduction

Ocular melanoma represents 5% of all cases of melanoma. Melanoma of the choroid is the most common malignant ocular tumor in adults, accounting for over 80% of cases. The global incidence is about four to eight cases per million per year. The incidence in North America and Europe varies between two to eight cases per million annually. In the United States the incidence is five to six cases per million. In Europe, Nordic countries reach the highest rates at four to five per million; however, Spain and Southern Italy achieve the lowest rates with two cases per million. Data from Africa and Asia show very low with incidence rates of 0.3 cases per million per year [1,2,3,4,5,6,7].

The majority of cases are asymptomatic, but they can sometimes be manifested as visual acuity reduction or visual field alteration [1,2].

Uveal melanoma has an elevated preference for hematogenous metastasis with up to 50% of patients developing metastasis within 15 years despite a successful local treatment [1]. The liver is the most common organ involved, and the survival rate for metastatic disease is poor [1,2,8]. Despite advances in diagnostic testing, uveal melanoma is associated with poor prognosis due to the lack of effective treatments of metastatic disease. However, histopathological study and molecular tumor analysis can provide interesting data about the metastatic risk. The prognostic factors are age, tumor size, tumor site, histologic type, mitotic number, vascular pattern, and tumor infiltrating lymphocytes. The presence of certain mutations in some genes such as the SF3B1, EIF1AX, and BAP1 genes also influences the prognosis and can be studied due to improvements in molecular biology and digital pathology [2,7,8,9,10,11,12]. Chromosomal 6p gain is associated with a good prognosis; however, 1p loss, 3 loss, and 8q gain are correlated with high mortality rates [13].

BAP1 gene mutation has a strong association with both the presence of uveal melanoma and an increased risk of metastasis [1,2,8,11,14]. The BAP1 protein is codified by the BAP1 gene which is located in the short arm of chromosome 3 (3p 21.1). This protein has deubiquitinase activity and a tumor suppressor function. The BAP1 protein is involved in the cell control cycle, DNA reparation, and accelerated apoptosis. The lack of nuclear expression and the mislocalization of the BAP1 protein can result in tumor development and/or progression to metastatic disease [8,15,16,17,18,19,20].

An immunohistochemistry technique which detects BAP1 protein alterations has been discovered [15,21]. The lost function can be caused by direct BAP1 gene mutations or other accessory gene mutations. The BAP1 immunohistochemistry is a sensitive technique based on the use of IgG1C4 monoclonal mice antibodies [22,23]. Its application on paraffin-embedded tissue has been shown to detect inhibited functions in BAP1 proteins. Moreover, protein inhibition indicates a worse prognosis due to its relationship with the presence of metastasis and death [8,15,18,19,21,23,24,25].

Uveal melanoma is not the only tumor that has been related to inhibited BAP1 proteins. Other tumors such as BAP-oma, cutaneous melanoma, mesothelioma, and/or renal cell carcinoma have also been related to this alteration and constitute the BAP1 related tumor predisposition syndrome [17,26].

The main objectives of this study were to discover the clinical and histological features of uveal melanoma in a regional Spanish community and to evaluate the application of BAP1 immunohistochemistry staining in this group. This analysis could help to show, indirectly, the presence or absence of BAP1 mutation, its relationship with other prognosis factors, and its effect on survival rates.

## 2. Material and Methods

### 2.1. Study Design

This was a retrospective observational study between January 1980 and December 2018 carried out in a tertiary referral hospital. The study was approved by both the ethics committee of Complejo Hospitalario de Navarra and the University of Navarre, in Pamplona, Spain. It was conducted in accordance with the principles of the Helsinki Declaration, and written informed consent was obtained from all the participants by the principal investigator. Appropriate exemptions were obtained for the deceased people with a certificate of discharge released by the president of the investigation commission of the Complejo Hospitalario de Navarra.

The criteria for inclusion were that the patient be 18 years or older and diagnosed with uveal melanoma in the Navarre sanitary region. The criterion for exclusion was any other type of ocular melanoma such as conjunctival or ocular adnexa melanoma, which was present in 2 patients.

Clinical features of 84 patients were evaluated by computerized medical records analysis. Although 46 patients received surgery, histopathological and immunohistochemical features were only evaluated in 40 enucleated eyes. This was due to six patients choosing to receive their operations in other hospitals making it impossible to obtain the samples.

The clinical information included patient age, sex, symptoms, laterality, tumor site, treatment received, presence and localization of metastasis, and death due to metastatic melanoma. The histopathological and immunohistochemical features included tumor size (largest basal diameter (LBD) and thickness), histologic type, vascular invasion, ocular structures invasion, growth pattern, mitosis, the presence of pigment, tumor infiltrating lymphocytes, necrosis, vascular pattern, pathologic stage, and immunohistochemical markers such as MelanA, Ki67, S100, HMB45, SOX10 and BAP1. The metastasis-free follow-up time was also recorded.

### 2.2. Histology

**Figure 1:**
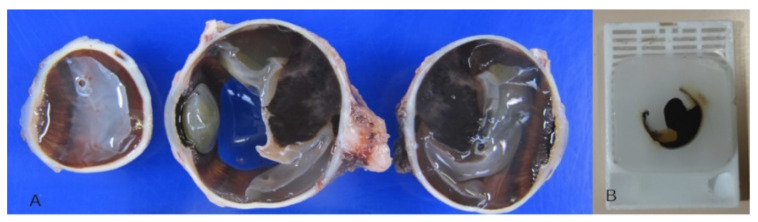
(**A**) Three slices of a formalin fixed enucleated eyeball with uveal melanoma. (**B**) An eyeball sample embedded in paraffin.

The enucleated eyes were fixed in formalin (10%) for at least 48 h, sliced into sections (Figure 1A), and embedded in paraffin (Figure 1B). Subsequently, 5 µm thick sections were prepared and mounted on glass slides for dewaxing with xylene. They were then rehydrated with ethanol and distilled water. The sections were stained with hematoxylin and eosin (H&E) according to usual protocols and assessed by two pathologists under a stereomicroscope with transmitted light.

### 2.3. Immunohistochemical Staining

MelanA, Ki67, S100, HMB45 and SOX 10 immunohistochemistry stains were performed according to routine protocols.

BAP1 immunohistochemistry staining was carried out according to the manufacturer protocol (sc-28383, 1:50 dilution, Santa Cruz Biotechnology, Dallas, TX, USA). After deparaffination, hydration, and heat-induced antigen retrieval, the sections were incubated with the BAP1 IgG1C4 antibody for 1 h at 36 °C. The combination with a red chromogen (Ultra view universal alkaline phosphatase detection kit; Ventana) was very important to avoid obfuscation of immunoreactivity with melanin pigment.

All these steps were very important. If an error in the methodology of staining was shown, a new sample staining was needed to avoid false positive or negative results. 

**Figure 2:**
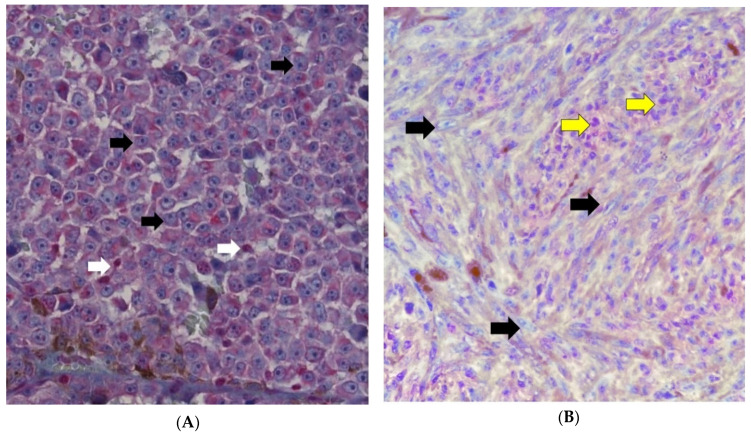

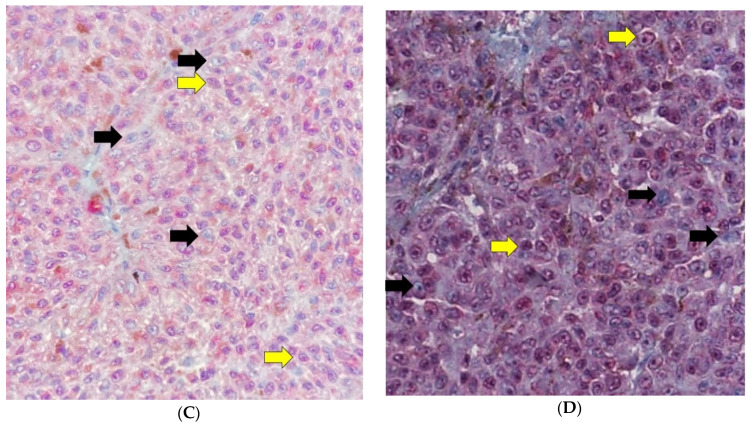
Nuclear BAP1 staining. (**A**) Grade 0 = positive staining in less than 10% of cells per high-power field (×400). Negative nuclear stain (black arrows) and lymphocytes used as positive control (white arrow). (**B**) Grade 1 = positive staining between 10% and 33% of cells per high-power field (×400). Negative nuclear stain (black arrows) and positive nuclear stain (yellow arrows). (**C**) Grade 2 = positive staining between 34% and 66% of cells per high-power field (×400). Negative nuclear stain (black arrows) and positive nuclear stain (yellow arrows) (**D**) Grade 3 = positive staining in ≥66% of cells per high-power field (×400). Negative nuclear stain (black arrows) and positive nuclear stain (yellow arrows).

**Figure 3:**
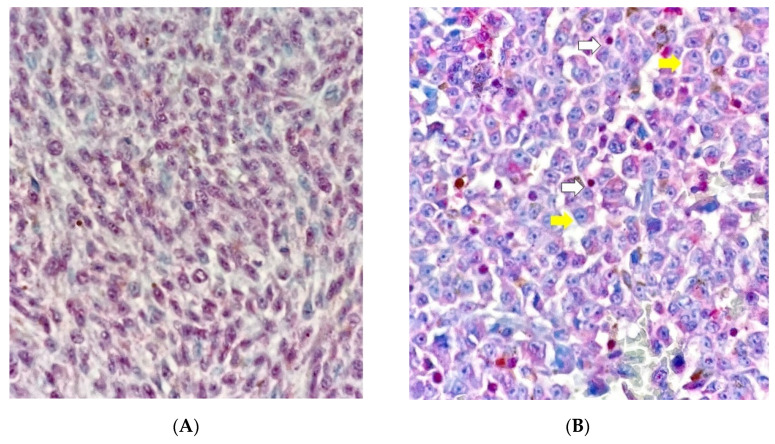

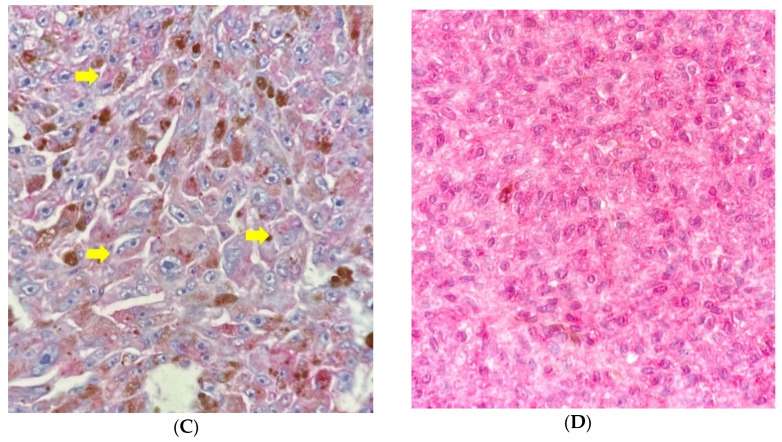
Cytoplasmic BAP1 staining. (**A**) Grade 0 = positive staining in less than 10% of cells per high-power field (×400). (**B**) Grade 1 = positive staining between 10% and 33% of cells per high-power field (×400). Positive cytoplasmic stain (yellow arrows) and lymphocytes used as positive control (white arrow). (**C**) Grade 2 = positive staining between 34% and 66% of cells per high-power field (×400). Positive cytoplasmic stain (yellow arrows). (**D**) Grade 3 = positive staining in ≥66% of cells per high-power field (×400).

The most intense BAP1 stained areas were selected for grading under 40× magnification. The samples with nuclear (Figure 2A–D) and cytoplasmic staining were separated from each other (Figure 3A–D) using the following scoring system: 0 = positive staining in less than 10% of cells per high-power field, 1 = positive staining in between 10% and 33% of cells per high-power field, 2 = positive staining in between 34% and 66% of cells per high-power field, and 3 = positive staining in ≥66% of cells per high-power field. Positive BAP1 immunoreactivity was shown by an increase in red staining in the cellular nucleus or cytoplasm (Figure 2D and Figure 3D); while negative BAP1 immunoreactivity was shown by a decrease in red staining which caused the cells to have a blue appearance (Figure 2A and Figure 3A). The retinal pigment epithelium and the lymphocytes were used as positive controls for BAP1 expression (Figure 2A white arrows).

Another BAP1 mouse monoclonal antibody is available (BAP1 IgG1 Clone BSB-109 of Bio SB Inc., Goleta, CA, USA) but there are not enough cases in the literature on uveal melanoma to compare our results.

All tissues embedded in paraffin were well preserved despite the passage of time; so, histology and immunohistochemical staining were performed and analyzed without any impairment.

An ophthalmologist and two pathologists evaluated the histopathological and immunohistochemical features in a blinded manner. If a heterogeneity of staining, due to a processing sample error was noted, the test was repeated.

**Figure 4:**
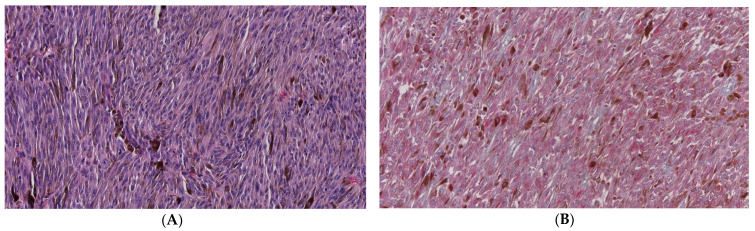
Uveal melanoma sample. (**A**) H&E image (×200). (**B**) Grade 0 of nuclear BAP1 staining with grade 3 of cytoplasmic BAP1 staining (×200).

H&E image (Figure 4A) and grade 0 of BAP1 nuclear staining with grade 3 of BAP1 cytoplasmic staining image (Figure 4B) in the same sample of UM are shown.

### 2.4. Statistics

Descriptive statistics were calculated on demographic, clinical, histological, and immunohistochemical factors for patients with uveal melanoma.

Data were recorded and added to IBM SPSS Statistics 25 for analysis. The Shapiro–Wilk and the Kolmogorov–Smirnov tests were employed to check normality.

Results were expressed as either mean and standard deviation (SD) or median and range if the variables are continuous, or they were expressed as frequencies and percentages if the variables were categorical. The following baseline variables were compared: enucleated versus non-enucleated patients, the presence or absence of metastasis, and the different grades of nuclear and cytoplasmic BAP1 stain. A Student’s *t*-test was used in continuous variables with normal distribution such as age and tumor thickness, and Mann–Whitney U test was performed in continuous variables with abnormal distribution such as LBD and number of mitosis. Chi square or Fisher tests were used for categorical variables.

Moreover, metastasis-free survival (defined as the time from the diagnosis until the development of metastatic disease) was analyzed using Kaplan–Meier method. The Log Rank Test was used to analyze the metastasis-free survival based on the nuclear or cytoplasmic BAP1 stain grade.

Statistical significance was set at *p* < 0.05.

## 3. Results

Eighty-four patients (49 males and 35 females) diagnosed with primary uveal melanoma in Navarre between January 1980 and December 2018 were included in this analysis. The mean age was 63.4.

Forty-four patients were enucleated, and two cases were exenterated.

Clinical data were collected for forty-six patients with enucleation and/or exenteration. Thirty-nine patients presented with symptoms, with reduced visual acuity and scotoma being the most frequent. Two patients were treated with pars endoresection, and four patients received brachytherapy before enucleation. Metastases were present in twenty patients (43.5%) (eighteen with liver metastasis (90%), nine with lung metastasis (45%), and three with bone metastasis (6.5%)). Sixteen patients (80%) died due to metastatic uveal melanoma.

**Figure 5:**
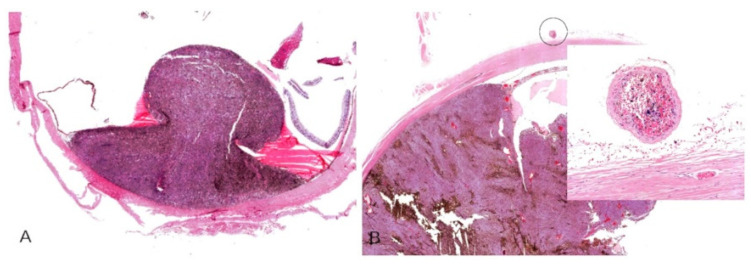
(**A**) H&E image (panoramic view): Uveal melanoma with mushroom-shape. (**B**) H&E image (×40): Vascular invasion, malignant cells in a vorticose vein (×400).

A clinicopathological study was performed on forty enucleated eyeballs. The tumors had a mean LBD of 11.57 mm ± 3.85 mm, and a mean height of 8.02 mm ± 3.4 mm. Retinal detachment was present in 90% of patients. The mixed cell type was found in 43.6% of cases, and the epithelioid cell type was found in 15.4% of cases. Eighteen tumors were dome-shaped, thirteen had nodular forms, eight were mushroom-shaped (Figure 5A) and one tumor had a diffuse form. In the tumors, an arch vascular pattern was found in 51.4%, a parallel vessel pattern was found in 13.5%, and a network pattern was found in 10.8%. The pathological size was medium in 87.5% of cases. Vascular invasion was observed in three cases (Figure 5B). The sclera was affected in eleven cases and the ciliary body was affected in three cases. The optic nerve was established as surgical margin, and it was affected in two cases.

The clinicopathological features of enucleated eyeballs with or without metastasis are shown in Table 1. Significantly higher LBD tumor (*p* < 0.001), tumor infiltrating lymphocytes (*p* = 0.020), higher pathological staging (*p* = 0.042), and lower nuclear BAP1 stain (*p* = 0.001) were observed in the metastatic group.

**Figure 6:**
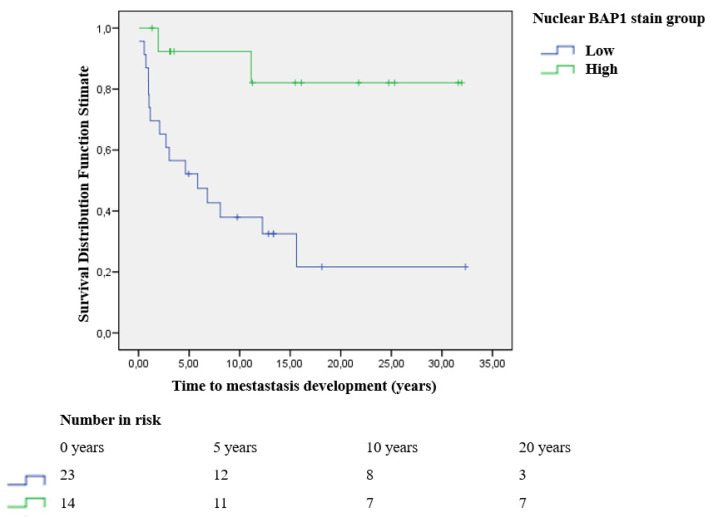
Kaplan–Meier metastasis-free survival curve in uveal melanoma patients with low and high grade nuclear BAP1 stain (*p* = 0.003).

BAP1 protein expression was analyzed in 40 eyeballs. It was not possible in three cases due to a high grade of tissue necrosis. Nuclear BAP1 staining was low (grade 0–1) in 23 patients and high (grade 2–3) in 14 patients. A significant association was seen between low nuclear BAP1 staining and death due to metastatic UM (*p* = 0.006), the presence of metastasis (*p* = 0.001), the presence of metastasis in the liver (*p* = 0.001), and a higher LBD (*p* = 0.01). Moreover, long term metastasis-free survival was higher in patients with high nuclear BAP1 staining (*p* = 0.003) (Figure 6).

Cytoplasmic BAP1 staining was also analyzed but no statistically significant differences were found.

## 4. Discussion

Despite the fact that the incidence of UM is not high, death due to the presence of distant metastasis remains elevated. Our study showed an incidence of 3.8 cases per million per year. This is higher than the Spanish incidence rate but is quite similar to the European incidence rate [3,4,5,6]. Kashyap S et al. noted that the incidence rate increased with age [27], the sixth and seventh decades being the most affected [28]. In our study we noted a similar increase in those patients (52.38%).

One of the main objectives in studying UM is to understand the intrinsic mechanisms which lead to metastatic disease. Chromosome alterations, such as loss of 1p, 3, 6q, and 8p or gain of 1q, 6p, and 8q, as well as some gene mutations, such as SF3B1, EIF1AX, are well known. However, several gene mutations and molecular bases are still under investigation. Barbagallo et al. noted LINC00518 as a new potential oncogene in UM [29]. Russo et al. established P16INK4a as an immunohistochemical expression in a large proportion of UM patients and liver metastasis due to alteration in CDKN2A gene [30]. This molecular background opens new possibilities in the diagnosis of melanoma, the study of metastasis, and treatment of UM.

The majority of studies show that 25–50% of UM cases will develop systemic metastasis [2,31,32]. At diagnosis, more than 95% of patients have eye limited disease, but death due to systemic metastasis reaches 30% by 5 years from the diagnosis and 45%, 50%, and 52% by 15, 25, and 35 years, respectively [31,32,33]. In our study, 28.6% of patients developed metastatic disease, and death due to systemic disease reached 53.1% (95% confident interval (CI) 34.74–70.91), 59.4% (95% CI, 40.64–76.30) and 62.5% (95% CI, 43.69–78.9) by 5, 15, and 20 years from the diagnosis, respectively, according to cumulative incidence analysis. The leading death cause in the majority of studies was metastatic uveal melanoma [32,34,35,36,37]. This was also the main cause of death in our research (62.5%). Furthermore, of the total of patients who died due to uveal melanoma, 85% did so within 5 years of diagnosis (Table 2).

Hematogenous dissemination is the main way that UM spreads, with the liver being the preferred metastatic site [31,33,34,35,38], as our study has corroborated (91.6%). Although only 5% of patients present with metastasis at diagnosis [33,34,35], the later liver involvement could indicate an undetectable spreading neoplasm that may be present at the time that the primary tumor is diagnosed. In our study, only one patient presented with systemic disease when diagnosed; however, twenty-four patients developed metastasis by their follow-up time. After the detection of metastasis, the prognosis is poor. The median survival range is between 2 to 24 months [1,2,8,28,32,34,37,39] with a median of 2.23 months in our patients. For this reason, prediction of metastasis is crucial for the patient’s prognosis, and the aim of this study is to confirm the most significant risk factors for metastatic dissemination such as BAP1 mutation.

To the best of our knowledge, it is the first time that nuclear BAP1 stain was evaluated as a survival predictor in UM patients in Spain. BAP1 gene mutations have a strong association with an absent BAP1 protein expression [8,15,23], and in addition, the lack of protein expression and mislocalized protein expression result in tumor progression, the appearance of metastasis, and reduced rates of survival [1,2,8,9,11,15,19,22,40]. Although effective systemic treatments have still not been established, some studies show that patients feel an uncertain prognosis is more stressful than a poor one. A clearly defined prognosis allows special attention to be guided to high-risk patients [41].

BAP1 immunohistochemistry also has an important role in the diagnosis and classification of malignant pleural mesothelioma, epithelioid atypical Spitz tumors, cutaneous melanoma, and clear cell renal cell carcinoma. It is a sensitive and specific method for the assessment of BAP1 inactivation or mislocalization and helps the pathologist to identify high-risk tumors [42,43,44].

About 62–84% of BAP1 somatic mutations have been identified in metastatic uveal melanoma [1,8,10,22,40,45]. In our study, BAP1 immunohistochemical staining was performed to evaluate the relationship between BAP1 protein expression and the development of metastasis. Although the age of the tissue samples and slides is a factor that influences BAP1 staining [22], all the samples in our study were re-stained when necessary. A red chromogen was used to avoid obfuscation of immunoreactivity with the melanin pigment, and the retinal cells were used as internal positive control, like in the study conducted by Szalai E et al. In some cases, heterogeneity of staining was noted, and the test was repeated to avoid this. Only three cases could not be analyzed due to necrotic tissue.

**Figure 7:**
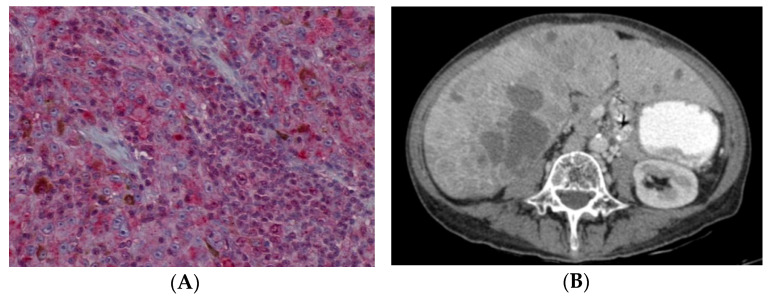
(**A**) H&E image (×200): Low grade nuclear BAP1 staining in a patient who died due to uveal melanoma metastasis. (**B**) Liver metastasis in an axial slice of a computed tomography scan.

Metastatic rates were different between patients with low grade and high grade nuclear BAP1 stain (*p* < 0.001). Low grade stain was recorded in sixteen metastatic UM patients (88.9%) and in seven non-metastatic UM patients (36.8%). By contrast, only two patients with a high grade nuclear BAP1 stain showed metastasis (14.3%). This was comparable with other studies in which lower nuclear immunoreactivity was observed in patients with a high risk of metastatic UM [1,8,15,19,25,40]. Moreover, death due to metastasis was greater among patients with lower nuclear BAP1 stain (92.9%) (Figure 7A,B) than in patients with higher nuclear BAP1 stain (20%). The liver was the most affected organ in both grades (65.2% in lower grade and 7.1% in higher grade). Furthermore, survival analysis demonstrated a significant association between low grade staining and reduced survival time.

It is not clear if the location of the BAP1 protein in the cell interferes with the tumor suppressor function [1,11,15,20,21]. Kalarai et al. concluded that there was no relationship between cytoplasmic BAP1 stain grade and the presence of metastasis. Our research supported this conclusion, but more high-quality studies should be performed. By contrast, in malignant pleural mesothelioma, the presence of cytoplasmic BAP1 stain was associated with a favorable prognosis [43].

BAP1 mutations have also been associated with a cancer syndrome known as BAP1 cancer predisposition syndrome. UM, malignant mesothelioma, cutaneous melanoma, and renal cell carcinoma are the main tumors involved in this syndrome. It seems that BAP1 germline mutations cause a predisposition to hereditary cancers which may be more aggressive and emerge earlier in life [17,44,46,47,48,49]. The available data about these associated tumors were explored, but no statistical significance was found. It is interesting to know that 20.2% of our patients also presented with adenomatous colon polyps, but no association with BAP1 mutations and these polyps has been identified in the literature.

Although some Collaborative Ocular Melanoma Study (COMS) research shows that the thickness measurement of fixed tumor tissue may vary from the clinical measurements [50], tumor height and LBD in our study could only be determined histologically after enucleation because there were no data about clinical size in any of our patients’ medical records.

The 7th edition of the American Joint Committee on Cancer’s (AJCC) posterior uveal melanoma classification and the Union for International Cancer Control (UICC) establish a pathological tumoral stage based on LBD, tumor height, ciliary body, and extraocular extension. In addition, COMS classifies uveal tumors as small, medium, or large based on their thickness and basal diameter [51,52]. Several researchers report tumor size as a prognosis factor with small melanoma being considered better than larger tumors [31,33,50,53]. In our study, in the metastatic group and in patients with lower grade nuclear BAP1 stain, LBD was found statistically significant. This was in contrast with the non-metastatic group and the patients with higher grade nuclear BAP1 stain. Moreover, the pathological stage was also significant in the metastatic group. This was in contrast with the non-metastatic group where the pathological stage was not significant. However, the tumor size as classified by COMS was not significant in our research. This could be due to the limitations of the study because of the small sample size or the retrospective analysis.

## 5. Conclusions

In conclusion, the correlation between nuclear BAP1 immunostaining, the development of metastasis, and patient survival could help us to stratify UM patients. This risk classification allows for closer follow-up of those patients with a lack of nuclear BAP1 staining. We believe that this technique should be implemented as routine in pathological examinations and that it could be an alternative to genetic studies as it is reasonably easy to apply and interpret. By contrast, cytoplasmic BAP1 stain has not shown to be a relevant factor in patient prognosis.

In this study, BAP1 staining was analyzed in enucleated eyeballs. It could be interesting to study BAP1 stain in other ways such as circulating uveal melanoma cells in peripheral blood to predict prognosis. Moreover, developing an artificial intelligence program that objectively quantifies the stain grade would be very useful to compensate for tumoral heterogeneity and interobserver variation.

## Figures and Tables

**Table 1:** Clinicopathological features of enucleated eyeballs with or without metastasis.

		UM Patients with Metastasis N = 19 (%)	UM Patients without Metastasis N = 21 (%)	*p*-Value
Age (years) *		65.84 (±14.09)	60.29 (±15.20)	0.240
Male sex		13 (68.4)	12 (57.1)	0.462
Retinal detachment		18 (94.7)	18 (85.7)	0.607
Largest basal diameter (mm) *		13.81 ± 3.73	9.54 ± 2.71	0.000
Tumor thickness (mm) *		9.10 (±3.59)	7.04 (±2.97)	0.055
Tumor shape	Mushroom	3 (15.8)	5 (23.8)	0.929
	Nodular	6 (31.6)	7 (33.3)	
	Dome	9 (47.4)	9 (42.8)	
	Diffuse	1 (5.3)	0 (0)	
Vascular pattern	Arches	8 (44.4)	11 (57.9)	0.709
	Parallels	4 (22.2)	1 (5.3)	
	Networks	2 (11.1)	2 (10.5)	
	Others **	4 (22.3)	5 (26.3)	
Cell type	Epithelioid	4 (21.1)	2 (10)	0.696
	Spindle	7 (36.8)	9 (45)	
	Mixed	8 (42.1)	9 (45)	
Lymphocytes		15 (78.9)	9 (42.9)	0.020
Vascular invasion		2 (10.5)	1 (4.8)	0.596
Necrosis		6 (31.6)	6 (28.6)	0.836
Surgical margin affected (optic nerve)		0 (0)	2 (9.5)	0.738
Pathological stage	pT1	2 (10.5)	5 (23.8)	0.042
	pT2	3 (15.8)	10 (47.6)	
	pT3	10 (52.6)	5 (23.8)	
	pT4	4 (21.1)	1 (4.8)	
Pathological size	Large	3 (15.8)	0 (0)	0.164
	Medium	15 (78.9)	20 (95.2)	
	Small	1 (5.3)	1 (4.8)	
Mitosis ***		3 (1–50)	2 (1–14)	0.313
Nuclear BAP1 staining	Low grade	16 (88.9)	7 (36.8)	0.001
	High grade	2 (11.1)	12 (63.2)	
Cytoplasmic BAP1 staining	Low grade	8 (44.4)	11 (55)	0.516
	High grade	10 (55.6)	9 (45)	

* Mean (standard deviation). ** Silent, straight, parallel-cross, arch-networks, straight-networks. *** Median (range).

**Table 2:** Percentage of deaths due to specific causes relative to all deaths during follow-up.

	Uveal Melanoma Mestastasis			No Evidence of Malignancy			Unknown			All Deaths	
Interval (years)	n	%	95% CI	n	%	95% CI	n	%	95% CI	n	%
0–5	17	77.27	54.63–92.18	2	9.09	1.12–29.16	3	13.64	2.9–34.91	22	68.75
5–10	2	50	6.76–93.24	2	50	6.76–93.24	0	0	0–60.24	4	12.5
10–15	0	0	0–84.19	0	0	0–84.19	2	100	15.81–100	2	6.25
15–20	1	100	2.5–100	0	0	0–97.5	0	0	0–97.5	1	3.125
>20	0	0	----	1	33.3	0.84–90.57	2	66.67	9.43–99.16	3	9.375
Total	20			5			7			32	

## Data Availability

Data supporting reported results can be found in Ophthalmology and Pathology Deparments of Complejo Hospitalario de Navarra, Pamplona, Navarra, Spain.

## Abbreviations

AJCC (American Joint Committee on Cancer); BAP1 (BRCA1-Associated Protein-1); CI (Confident Interval); COMS (Collaborative Ocular Melanoma Study); EIF1AX (Eukaryotic Translation Initiation Factor 1A X-Linked); GNAQ (Guanine nucleotide-binding protein G subunit alpha Q); GNA11 (Guanine nucleotide-binding protein G subunit alpha 11); HMB45 (HMB45 antibody); H&E (Hematoxylin and Eosin); IgG1C4 monoclonal mice antibodies (Immunoglobulin G1 beta actin antibody); Ki67 (Cell proliferation index Ki67); LBD (Largest basal diameter); MelanA (Melanoma Antigen); SD (Standard Deviation); SF3B1 (Splicing factor 3B subunit 1); SOX10 (SRY-related HMG-box 10 protein); S100 (S100 protein); UICC (Union for International Cancer Control); UM (Uveal Melanoma).
